# Supplementary figures and images for: Non-microtubule tubulin-based backbone and subordinate components of postsynaptic density lattices
Source: Life Sci Alliance. 2021 May 18;4(7):e202000945. doi: 10.26508/lsa.202000945 (PMC8326785; doi:10.26508/lsa.202000945)

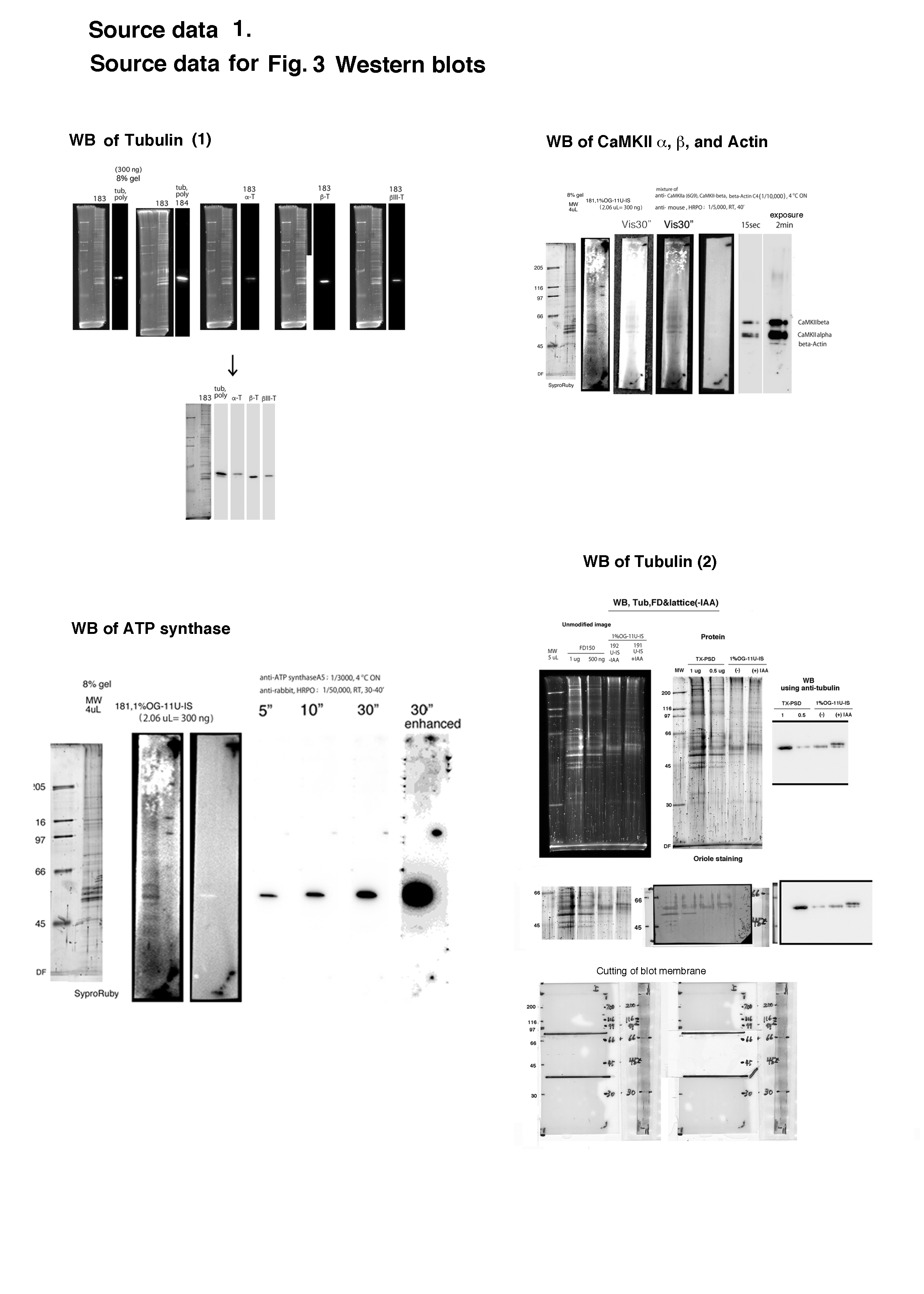

Supplement: Supplementary file 1 [file LSA-2020-00945_SdataF3.tif]

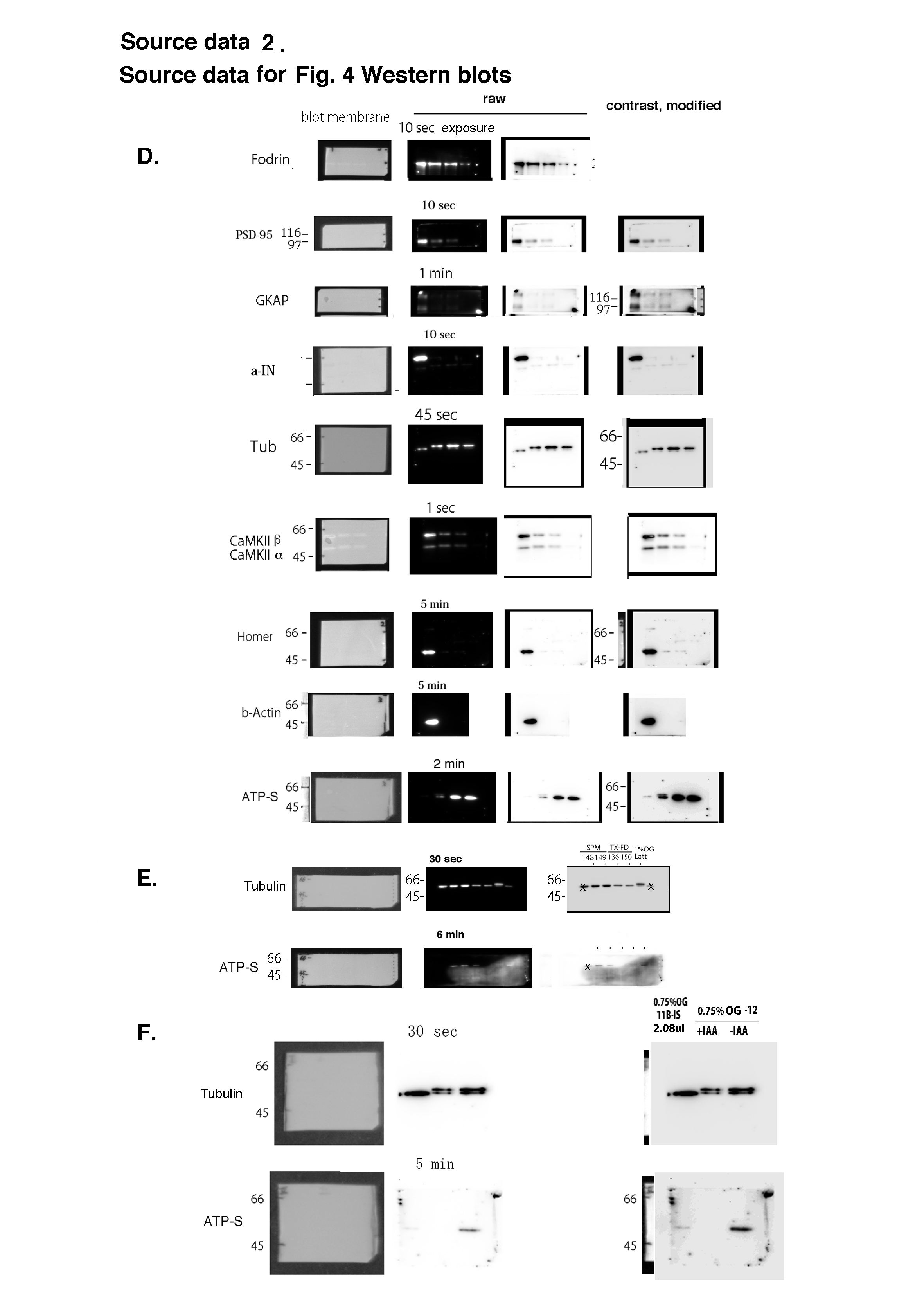

Supplement: Supplementary file 2 [file LSA-2020-00945_SdataF4.tif]

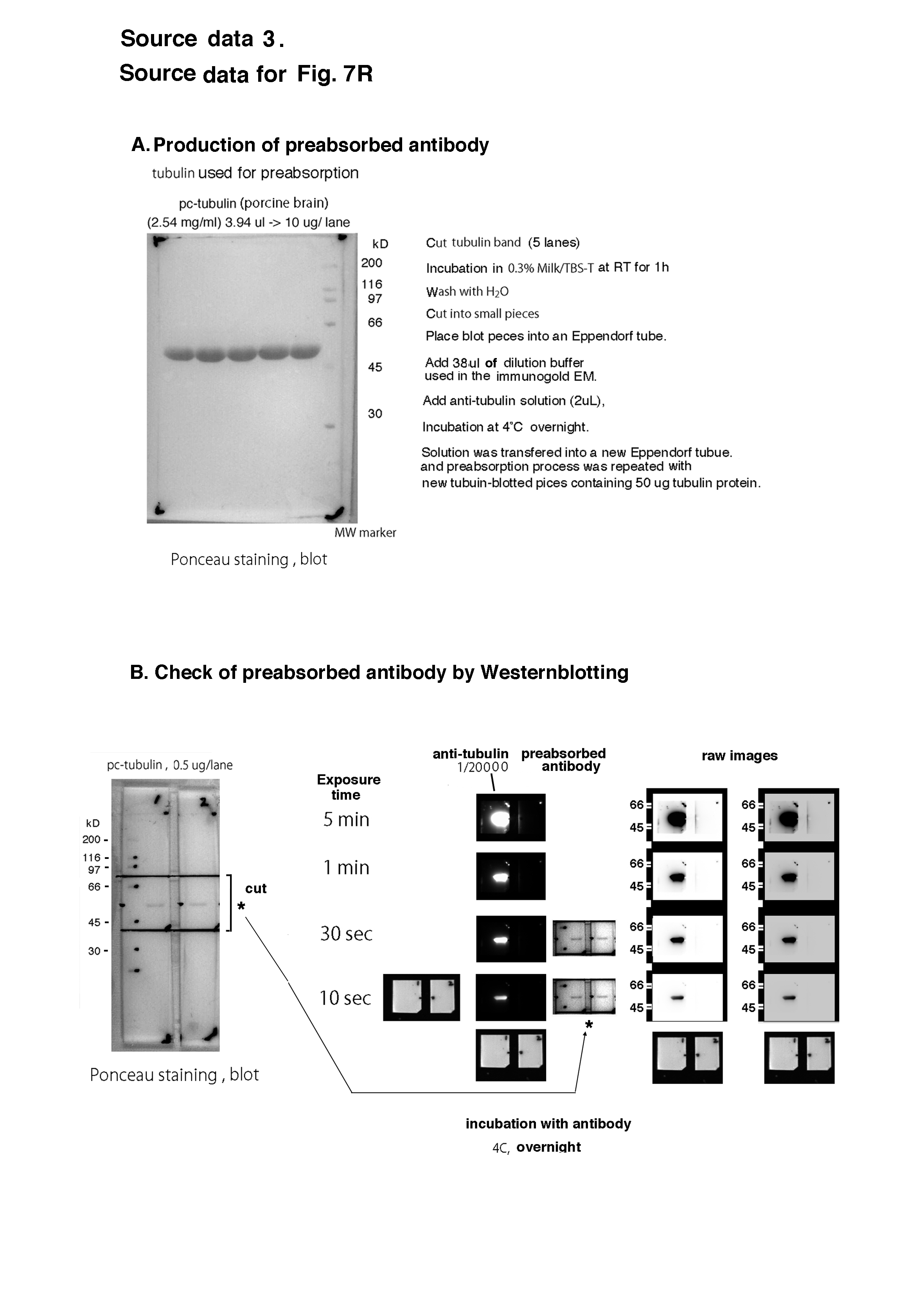

Supplement: Supplementary file 9 [file LSA-2020-00945_SdataF7.tif]
